# Molecular Characterization of an Aquaporin−2 Mutation Causing Nephrogenic Diabetes Insipidus

**DOI:** 10.3389/fendo.2021.665145

**Published:** 2021-08-27

**Authors:** Qian Li, Bichao Lu, Jia Yang, Chao Li, Yanchun Li, Hui Chen, Naishi Li, Lian Duan, Feng Gu, Jianmin Zhang, Weibo Xia

**Affiliations:** ^1^Department of Endocrinology, Key Laboratory of Endocrinology, NHC, State Key Laboratory of Complex Severe and Rare Diseases, Peking Union Medical College Hospital, Chinese Academy of Medical Sciences, Beijing, China; ^2^Department of Immunology, Research Center on Pediatric Development and Diseases, Institute of Basic Medical Sciences, Chinese Academy of Medical Sciences and School of Basic Medicine, Peking Union Medical College, State Key Laboratory of Medical Molecular Biology, Beijing, China; ^3^Department of Radiation Oncology, Stanford University, School of Medicine, Stanford, CA, United States

**Keywords:** aquaporin 2, nephrogenic diabetes insipidus, water reabsorption, polydipsia, hypernatremia

## Abstract

The aquaporin 2 (AQP2) plays a critical role in water reabsorption to maintain water homeostasis. AQP2 mutation leads to nephrogenic diabetes insipidus (NDI), characterized by polyuria, polydipsia, and hypernatremia. We previously reported that a novel *AQP2* mutation (G215S) caused NDI in a boy. In this study, we aimed to elucidate the cell biological consequences of this mutation on AQP2 function and clarify the molecular pathogenic mechanism for NDI in this patient. First, we analyzed AQP2 expression in Madin-Darby canine kidney (MDCK) cells by AQP2-G215S or AQP2-WT plasmid transfection and found significantly decreased AQP2-G215S expression in cytoplasmic membrane compared with AQP2-WT, independent of forskolin treatment. Further, we found co-localization of endoplasmic reticulum (ER) marker (Calnexin) with AQP2-G215S rather than AQP2-WT in MDCK cells by immunocytochemistry. The functional analysis showed that MDCK cells transfected with AQP2-G215S displayed reduced water permeability compared with AQP2-WT. Visualization of AQP2 structure implied that AQP2-G215S mutation might interrupt the folding of the sixth transmembrane α-helix and/or the packing of α-helices, resulting in the misfolding of monomer and further impaired formation of tetramer. Taken together, these findings suggested that AQP2-G215S was misfolded and retained in the ER and could not be translocated to the apical membrane to function as a water channel, which revealed the molecular pathogenic mechanism of AQP2-G215S mutation and explained for the phenotype of NDI in this patient.

## Introduction

Nephrogenic diabetes insipidus (NDI) is characterized by impaired arginine vasopressin (AVP)-induced water reabsorption in the kidney, leading to polyuria, polydipsia, and hypernatremia. The most severe outcomes include impaired mental development, dilation of the urinary tract, and death (1–3). NDI can be secondary to other clinical conditions, such as drugs (e.g., lithium and cisplatin therapy) and electrolyte abnormalities, or caused by mutations in the vasopressin V2 receptor (*AVPR2*, OMIM#304800) or *AQP2* (OMIM#125800, 107777) (4–7). AVPR2 accounts for X-linked cases of NDI. Current therapeutic options for congenital NDI focus on ameliorating symptoms rather than curing, which are limited and only partially effective (3).

AQP2 is a key factor for maintaining normal body water homeostasis. When the plasma osmolality increases, antidiuretic hormone (AVP) is released from the pituitary gland and binds to AVPR2 in principal cells of the kidney collecting duct, resulting in the accumulation of AQP2 in the apical plasma membrane, which is responsible for water reabsorption (8, 9). AQP2 forms a homotetramer in the plasma membrane, and each monomer is composed of 271 amino acids, containing six transmembrane spanning regions with the intracellular COOH terminus, which is essential for correct routing of AQP2. AVP increases phosphorylation of AQP2 at ser256 and ser269, which is important for the accumulation of AQP2 (10–12). K63-linked ubiquitylation of lys270 is critical for the internalization and degradation of AQP2 from the plasma membrane (13). Up to now, there were 65 mutations of *AQP2* reported to cause NDI, and missense/nonsense mutation is the most common mutation type (14). More than 90% of mutations are inherited in autosome recessive mode, which can be categorized into three types, depending on the structural analysis: (i) the pore features (e.g. A70D), (ii) the tetramer assembly (e.g. T126M), and (iii) the monomer folding (e.g. A47V). The remaining 10% of mutations are inherited in a dominant trait, involving the C-terminal tail for AQP2 routing (e.g. R254L) (15–19). Most autosomal recessive cases had severe phenotypes in contrast to autosomal dominant NDI (20).

We previously reported a homozygous missense mutation AQP2-G215S (substitution of Gly215 with Serine) caused NDI in a boy for the first time (21). The initial symptom occurred at 4 months, and the male patient had a total urine volume greater than 4 L in 24 h when diagnosed. Sequence alignment of AQP2 proteins indicated Gly215 showed a 100% conservation among six different species. In the study, we aimed to elucidate the cell biological consequences of this mutation on AQP2 function and clarify the molecular pathogenic mechanism for NDI in this patient.

## Materials And Methods

### Cell Culture

MDCK cell line was obtained from Cell Resource Center, Basic Medicine Institute, Chinese Academy of Medical Sciences. MDCK cells were cultured in Eagle’s Minimum Essential Medium with 10% fetal bovine serum. All experiments were performed with the approval of the institutional review board and ethics committee of PUMCH, and written informed consents were obtained from the patients.

### Plasmid Construction

Coding sequence of human AQP2 was cloned into a vector containing the pCMV6 promoter. Mutant AQP2-G215S plasmid was constructed by site-directed mutagenesis (QuikChange Site-Directed Mutagenesis Kits, Agilent) and confirmed by sequence analysis.

### Electroporation

Cells were resuspended in 100 μl buffer, and 2 μg plasmid was added. Cells were transferred to a sterile 0.2-cm cuvette (Cell Line Nucleofector™ Kit L, Lonza) and electroporated using Lonza^®^ Nucleofector^®^ II electroporation system according to the manual and protocol. After transfection, cells were gently resuspended in pre-warmed medium. Indomethacin (5 × 10^−5^ M, Selleck) was added to culture medium 24 h after electroporation and incubated overnight. The medium was replaced with fresh medium containing indomethacin (5 × 10^−5^ M, Selleck) plus forskolin (5 × 10^−5^ M, Selleck) or not and incubated for 2 h. Then, the cells were harvested for protein extraction or fixed for immunocytochemistry.

### Western Blot

Total cellular proteins were extracted using RIPA buffer containing protease and phosphatase inhibitors. Total membrane and cytoplasmic membrane protein were extracted by a kit (Minute™ Plasma Membrane Protein Isolation and Cell Fractionation Kit, invent BIOTECHNOLOGIES) according to the protocol. All procedures were performed on ice, and protease and phosphatase inhibitors were added into buffer A before use. 20 to 50 × 10^6^ cells were prepared for plasma membrane protein isolation. Equal amounts of protein were loaded on a 12% SDS-polyacrylamide gel and transferred to nitrocellulose membranes. Membranes were blocked with 5% non-fat dry milk for 2 h, followed by incubation with primary antibodies (AQP2 polyclonal antibody, 1:1000, cell signaling technology, #3487; Cadherin antibody, 1:1000, Cell Signaling Technology, ab16505; Calnexin, 1:1000, Thermo Fisher Scientific, MA3-027) over night. Membranes were washed and incubated with HRP-conjugated goat anti-rabbit IgG. Bands were visualized by enhanced chemiluminescence (Pierce™ ECL Western Blotting Substrate kit, Thermo Fisher Scientific).

### Immunocytochemistry

Cells were fixed in 4% paraformaldehyde in PBS (pH 7.4) and blocked with 5% normal goat serum and 0.2% Triton X-100. Primary antibodies, including Calnexin (Thermo Fisher Scientific), were diluted in 5% normal goat serum and incubated overnight. Alexa Fluor 594- and Alexa Fluor 488-labeled secondary antibodies were used. Samples were counterstained with 4′,6-diamidino-2-phenylindole (DAPI) and mounted on glass slides using the ProLong antifade kit (Thermo Fisher Scientific).

### Transcellular Osmotic Water Permeability Measurements

Cells derived from 0.33 cm^2^ of confluent monolayers were seeded onto 0.33-cm^2^ polycarbonate filters (Costar, Cambridge, U.S.A.). On the second day after seeding, the medium was aspirated and replaced by fresh medium in the presence of indomethacin (5 × 10^−5^ M, Selleck) to reduce basal intracellular cyclic adenosine monophosphate (cAMP) levels. Three days after seeding, osmotic water transport was assayed in the presence of indomethacin with or without adding forskolin (5 × 10^−5^ M, Selleck), by incubation of the apical compartment with 150 µl of 0.5× KHB (1× KHB contains 1.2 mM MgSO_4_, 128 mM NaCl, 5 mM KCl, 2 mM NaHPO4, 10 mM NaAc, 20 mM HEPES, 1 mM CaCl_2_, 1 mM l-alanine, 4 mM L-Lactate; pH=7.4), containing 30 mg/L phenol-red and the addition of 800 µl KHB to the basal compartment. After incubation for 2 h at 37°C, the content of the apical compartment was mixed with a pipette, and two aliquots of 50 µl per insert were put into Eppendorf tubes and diluted to 600 µl with Tris-buffered saline (TBS: 20 mM Tris, 73 mM NaCl; pH=7.6) containing 1% (w/v) extrane (Merck, Darmstadt, Germany). After mixing and centrifugation, absorbance at 479 nm was measured. The osmotic water transport (Pf) was calculated from the acquired absorbances as described (22, 23).

### Visualization of AQP2 Structure

To visualize the potential impact of the G215S mutation, we built the crystal structure of G215S tetramer using homology modelling with Prime (24). The template structure was human Aquaporin 2 (PDB ID: 4NEF) with 99% sequence identity (17). The snapshots were prepared using VMD 1.9.3 (25).

### Data Analysis and Statistics

All results represented at least three independent replications. All data were represented as mean ± SEM. Statistical analysis was performed using GraphPad Prism software (San Diego, California USA, www.graphpad.com). Differences between groups were analyzed as appropriate using *t* test or one-way ANOVA and post-hoc Tukey’s multiple comparison tests. *P* < 0.05 (two tailed) was considered to be statistically significant.

## Results

### Expression of AQP2-G215S Was Decreased in the Cell Membrane Compared With AQP2-WT

**Figure 1:**
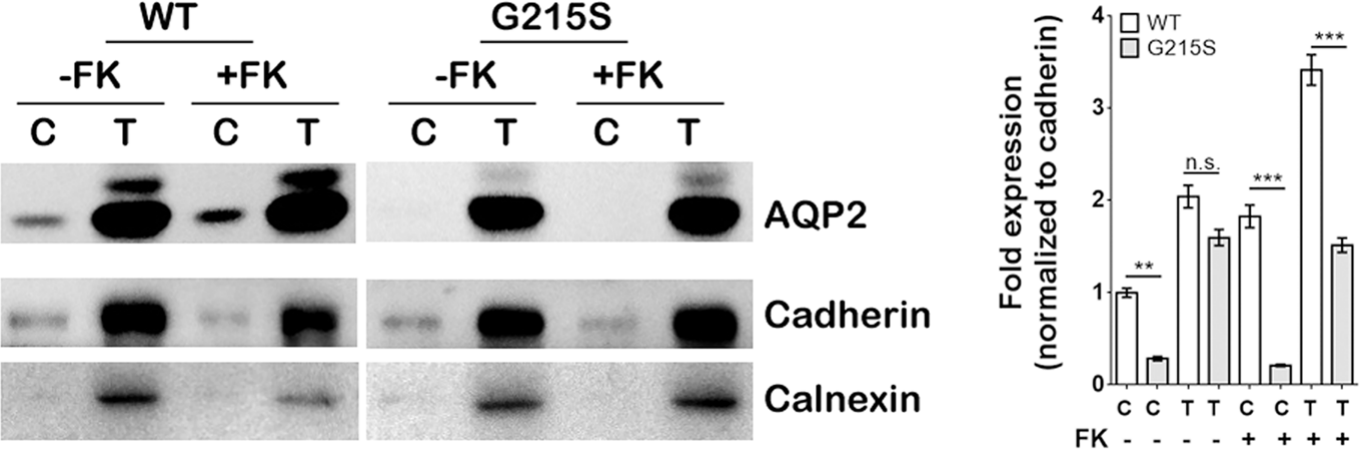
Expression of AQP2-G215S was decreased in the cell membrane compared with AQP2-WT. MDCK cells were transfected with AQP2-WT and AQP2-G215S plasmids. Total membrane proteins (labeled as T) and cytoplasmic membrane proteins (labeled as C) were harvested for AQP2, Pan-cadherin, and calnexin immunoblotting. Pan-Cadherin is the membrane marker, and calnexin is the endoplasmic marker. Protein fold expression normalized to cadherin is shown. WT, wild type; FK, forskolin. Data were shown as mean ± SEM. n = 3, **p < 0.01, ***p < 0.001, n.s.=no statistically significant difference.

We expressed AQP2-WT and AQP2-G215S in MDCK cells by electroporation with plasmid, a cell line demonstrated to be a typical model for AQP2 function (26). Since AQP2 is a membrane protein and functions as a water channel in the apical membrane of principle cell, we further isolated the cytoplasmic membrane and total membrane and detect AQP2 expression by WB (as shown in **Figure 1**). The results showed that the expression of AQP2 was similar in the total membrane of AQP2-WT-transfected cells and AQP2-G215S-transfected cells (p=0.44). However, AQP2 was decreased in the cell membrane of AQP2-G215S–transfected cells compared with AQP2-WT-transfected cells (p<0.01), even after treated with forskolin (p<0.001), suggesting that mutant AQP2-G215S may not be transported to the cell membrane.

### AQP2-G215S Is Retained in Endoplasmic Reticulum

**Figure 2:**
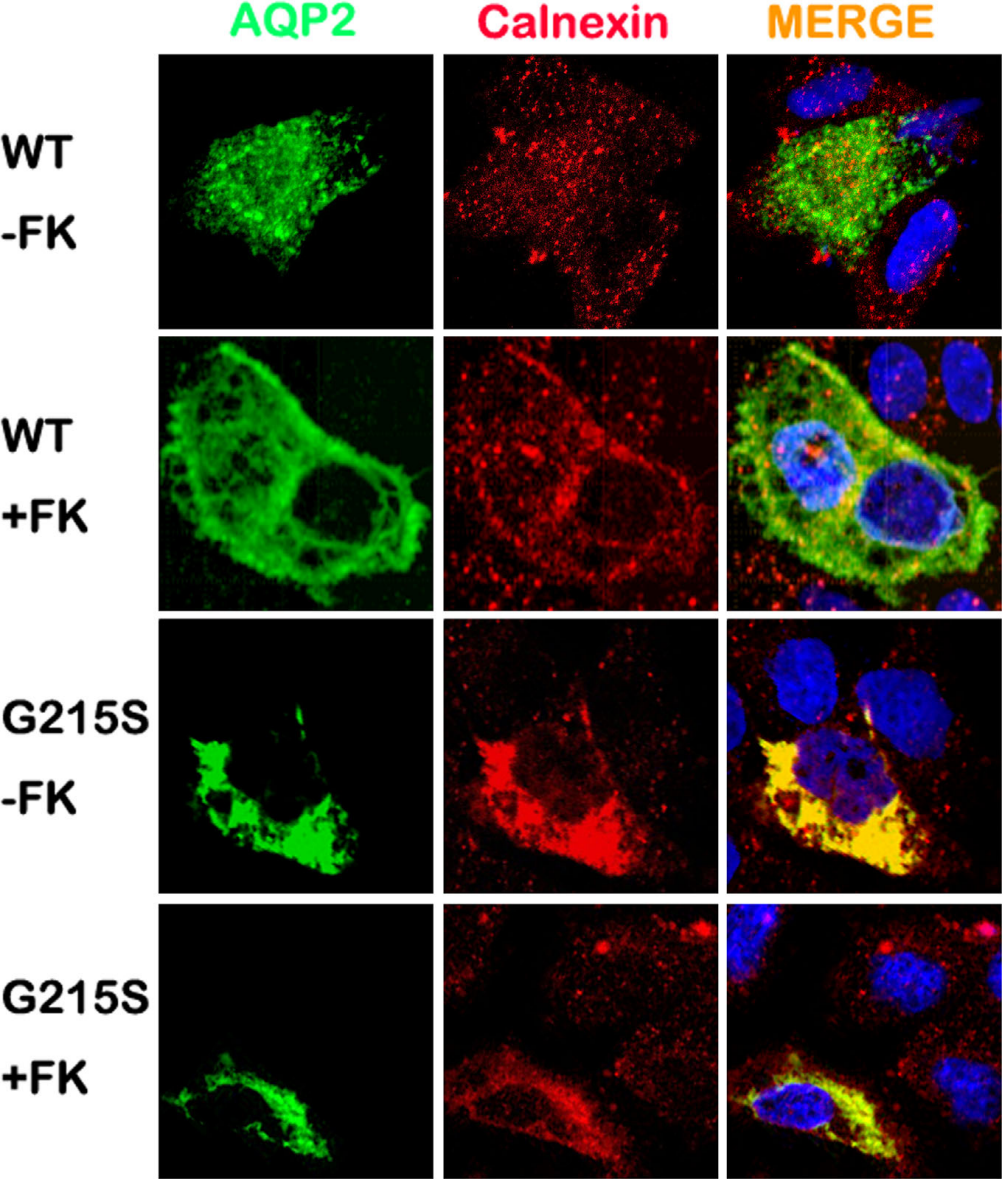
AQP2-G215S was retained in endoplasmic reticulum. MDCK cells were electroporated with AQP2-WT and AQP2-G215S plasmids and stained for AQP2 and endoplasmic reticulum marker (Calnexin). WT, wild type; FK, forskolin.

We further analyzed the subcellular localization of AQP2-G215S in MDCK cells by immunocytochemistry. MDCK cells were electroporated with AQP2-WT or AQP2-G215S plasmid, then fixed and stained to detect the expression of AQP2 and ER marker (Calnexin). Our results showed co-localization of AQP2 with Calnexin in AQP2-G215S-transfected cells compared with AQP2-WT-transfected cells with or without the stimulation of Forskolin (**Figure 2**), which suggested that AQP2-G215S was retained in endoplasmic reticulum, in contrast to AQP2-WT.

### AQP2-G215S Displays Impaired Transcellular Osmotic Water Permeability

**Figure 3:**
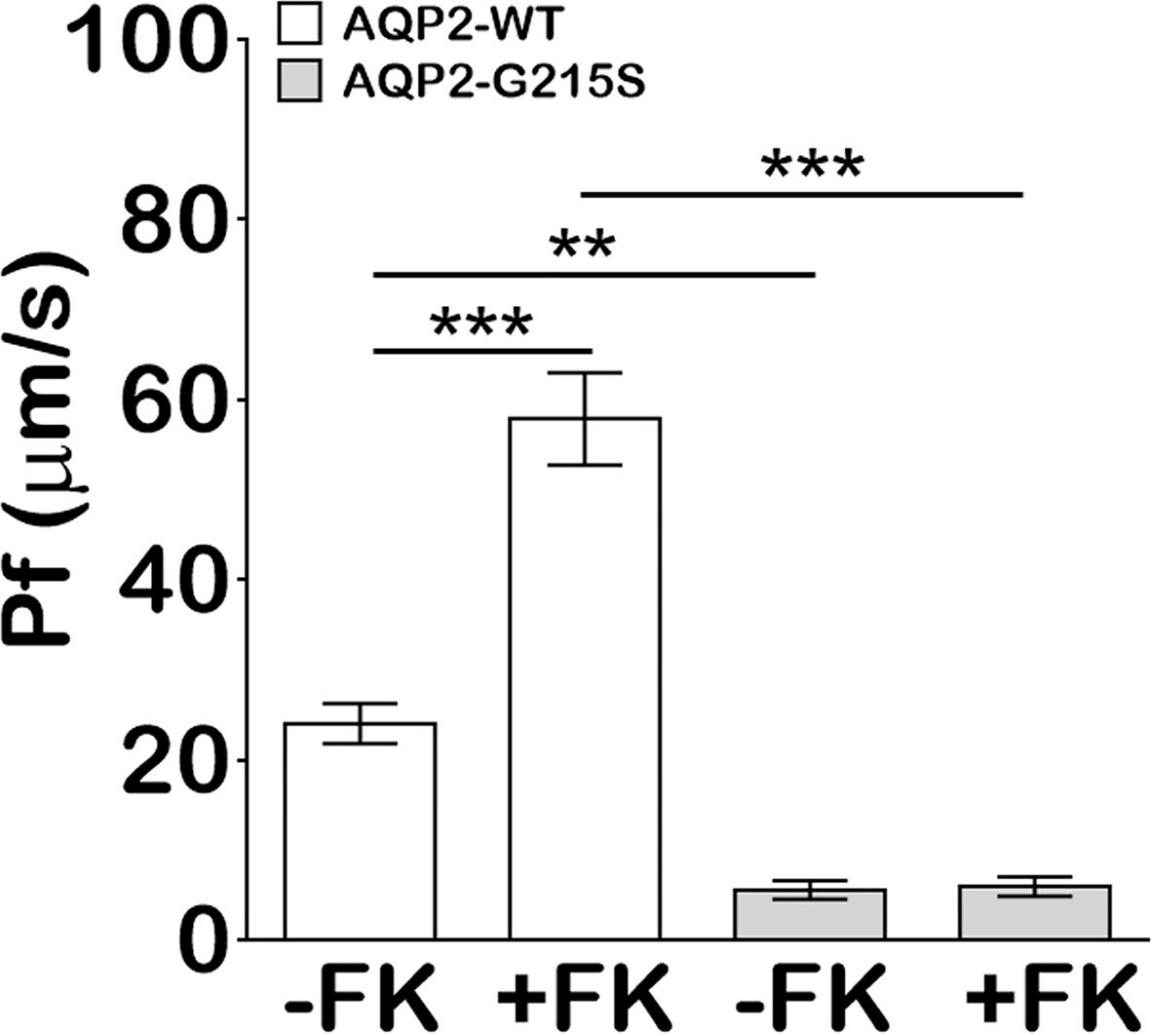
MDCK cells transfected with AQP2-G215S displayed impaired transcellular osmotic water permeability. Cells were seeded onto 0.33 cm^2^ polycarbonate filters, and replaced with fresh medium in the presence of 5 × 10^−5^ M indomethacin. Osmotic water transport was assayed with or without 5 × 10^−5^ M forskolin. The osmotic water transport (Pf) of AQP2-G215S-transfected and AQP2-WT-transfected cells was calculated as described in Material and Methods. WT, wild type; FK, Froskolin. Data were shown as mean ± SEM. n = 3, **p < 0.01, ***p < 0.001.

We analyzed the transcellular osmotic water permeability of AQP2-G215S- and AQP2-WT-transfected MDCK cells. The osmotic water transport (Pf) of AQP2-WT-transfected cells was higher than AQP2-WT–transfected cells (24 ± 2.2 *vs* 5.5 ± 1.0 μm/s, *p*<0.01) (**Figure 3**). With the stimulation of forskolin, the Pf of AQP2-WT-transfected cells was increased (57.8 ± 5.1 μm/s, *p*<0.001), whereas the Pf of AQP2-G215S did not change apparently and remained a much lower level than AQP2-WT (*p*<0.001). These results indicated that the permeability of MDCK cells transfected with AQP2-G215S was impaired, which further supported retention of AQP2-G215S mutant in endoplasmic reticulum.

### Structural Visualization of AQP2−G215S

**Figure 4:**
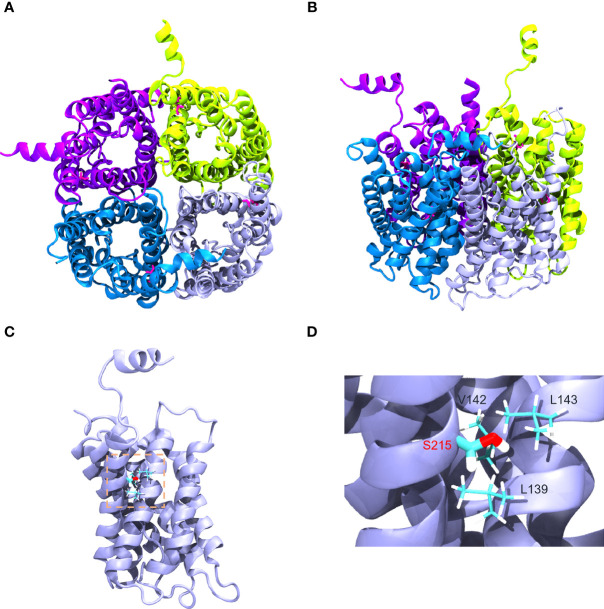
Structural visualization of AQP2−G215S. **(A, B)** The structure of AQP2 (PDB ID is 4NEF) was visualized as a tetramer. **(C, D)** Gly215 was indicated as red in the sixth membrane spanning alpha-helix of the AQP2 monomer as shown.

Gly215 was located at alpha-helix in the sixth transmembrane spanning of AQP2 monomer as shown in **Figures 4A** **, B**. Amino acids surrounding S215 were on the same monomer, including V142, L143, and L139, suggesting that G215S mutation mostly destabilized AQP2 monomer by changing interactions within intra-monomer helices (**Figure 4C**). Further, G215S, introducing a polar side chain of S215 into a hydrophobic environment, was thermodynamically unfavorable (**Figure 4D**). Based on these information, the serine substitution at Gly215 probably interrupted folding of the sixth transmembrane α-helix and/or the packing of α-helices, resulting in the misfolding of monomer, which may further influence the formation of functional AQP2 tetramer.

## Discussion

In this study, we aimed to elucidate cell biological consequences of a G215S mutation in AQP2 that was discovered in a boy with severe NDI (21). The AQP2 mutation was inherited from his parents, who had a heterozygous genotype and normal phenotypes, which supported an autosomal recessive inheritance model. There was a variety of cell lines and animal models applied to clarify molecular action of AQP2 and the pathogenic mechanism of NDI. MDCK, a kidney epithelia cell line, has been widely used as a model to study NDI, because AQP2 transfection reconstituted vasopressin-regulated transcellular osmotic water transport in principle cells of human renal collect duct (26–28). Here, we first constructed AQP2-G215S and AQP2-WT plasmids and analyzed the expression of AQP2-G215S and AQP2-WT by Western blot. Results showed that there was no difference of AQP2 in total membrane. AQP2 forms a homotetramer in the plasma membrane for water reabsorption, so we further isolated cell membrane of MDCK cells and found there was significantly decreased AQP2 expression in cell membrane of AQP2-G215S-transfected cells compared with AQP2-WT-transfected cells, which suggested that AQP2-G215S could not be translocated to cell membrane.

AQP2 monomer is a glycosylated membrane protein with six-pass-transmembrane domain, which is folded and assembled in the ER. During the processing, high-mannose sugar moieties are attached to Asn123 of AQP2, which is part of a canonical N-glycosylation consensus site (N123-X-T125), and the high-mannose sugar groups are removed in the Golgi complex en route to the plasma membrane (29, 30). To investigate the subcellular localization of AQP2-G215S and AQP2-WT in MDCK cells, immunocytochemistry was performed, and results showed co-localization of ER marker (Calnexin) with AQP2-G215S rather than AQP2-WT in MDCK cells with or without the stimulation of forskolin. These results suggested that the AQP2-G215S was retained in the ER, which was also reported in a previous study. For example, David et al. demonstrated that AQP2-F204V mutant was retained in the ER in a renal cell line and *in vivo* (31). Nannette also reported that most AQP2 missense mutants in recessive NDI are retained in the ER (32). There were other studies showing misrouting of AQP2 to Golgi complex (33) or late endosomes or lysosome (34). Here, we demonstrated mutant protein, AQP2-G215S, was retained in the ER for the first time. Functional analysis further demonstrated reduced water permeability in AQP2-G215S-transfected cells compared with AQP2-WT–transfected cells, which further supported that AQP2-G215S mutation was retained in the ER. We further performed structure analysis of AQP2-G215S mutant, and results showed that serine substitution at Gly215 probably interrupted the folding of the sixth transmembrane α-helix and/or the packing of α-helices, resulting in the misfolding of monomer, which may further influence the formation of functional AQP2 tetramer. There were also other studies aiming to elucidate the structural basis for mutations of human aquaporins. For instance, Luisa Calvanese built a 3D molecular model for AQP mutants and explored the effect of mutations on structural feature, which provided a rationale for interpreting mutations. Their results suggested that S216P caused impaired monomer folding, similar to G215S. Taken together, these results showed that AQP2-G215S mutant may be misfolded and retained in the ER and could not be translocated to the apical membrane to function as a water channel. There were limitations in our study, for example, cell biological consequences of this mutation were only investigated in MDCK cell line. Besides, novel therapy is further expected to improve the prognosis of NDI. Up to now, some chemical chaperones were shown to correct the trafficking and folding defects of AQP2 mutants and show efficacy in NDI treatment, and gene editing may correct such mutation and cure diseases in the future (35)

In conclusion, we investigated cell biological consequences of a novel mutation (AQP2-G215S) discovered in a boy with NDI. Results showed that AQP2-G215S mutant may be misfolded and retained in the ER and could not be translocated to the apical membrane to function as water channel. This knowledge elucidated the potential molecular mechanism for NDI in this patient.

## Data Availability Statement

The datasets presented in this study can be found in online repositories. The names of the repository/repositories and accession number(s) can be found in the article/supplementary material.

## Ethics Statement

Written informed consent was obtained from the minor(s)’ legal guardian/next of kin for the publication of any potentially identifiable images or data included in this article.

## Author Contributions

Study design: FG, JZ, and WX. Study conduct: QL, BL, JY, and CL. Data collection: QL, BL, JY, and CL. Data analysis: QL, BL, JY, CL, YL, HC, NL, LD, FG, JZ, and WX. Data interpretation: QL, BL, JY, CL, YL, HC, NL, LD, FG, JZ, and WX. Drafting manuscript: QL. Revising manuscript content: QL, BL, JY, CL, HC, NL, LD, FG, JZ, and WX. Approving final version of manuscript: QL, FG, JZ, and WX. All authors contributed to the article and approved the submitted version.

## Funding

This work was supported by National Natural Science Fund (No. 81670814), National Key R&D Program of China (2018YFA 0800801), and National Natural Science Fund (No. 81970757).

## Conflict of Interest

The authors declare that the research was conducted in the absence of any commercial or financial relationships that could be construed as a potential conflict of interest.

## Publisher’s Note

All claims expressed in this article are solely those of the authors and do not necessarily represent those of their affiliated organizations, or those of the publisher, the editors and the reviewers. Any product that may be evaluated in this article, or claim that may be made by its manufacturer, is not guaranteed or endorsed by the publisher.
